# A Colorimetric Aptasensor for Ochratoxin A Detection Based on Tetramethylrhodamine Charge Effect-Assisted Silver Enhancement

**DOI:** 10.3390/bios13040468

**Published:** 2023-04-10

**Authors:** Xiaoyan Yang, Rong Huang, Lulu Xiong, Feng Chen, Wei Sun, Ling Yu

**Affiliations:** 1 Key Laboratory of Luminescent and Real-Time Analytical Chemistry, Ministry of Education, Institute for Clean Energy and Advanced Materials, School of Materials and Energy, Southwest University, Chongqing 400715, China; 2 College of Chemistry and Chemical Engineering, Hainan Normal University, Haikou 571158, China

**Keywords:** aptamer, ochratoxin A, gold nanoparticle, silver enhancement, colorimetric aptasensor

## Abstract

A novel colorimetric aptasensor based on charge effect-assisted silver enhancement was developed to detect ochratoxin A (OTA). To achieve this objective, gold nanoparticles (AuNPs), which can catalyze silver reduction and deposition, were used as the carrier of the aptamers tagged with a positively charged tetramethylrhodamine (TAMRA). Due to the mutual attraction of positive and negative charges, the TAMRA attracted and retained the silver lactate around the AuNPs. Thus, the chance of AuNP-catalyzed silver reduction was increased. The charge effect-assisted silver enhancement was verified by tagging different base pair length aptamers with TAMRA. Under optimized conditions, the as-prepared OTA aptasensor had a working range of 1 × 10^2^–1 × 10^6^ pg mL^−1^. The detection limit was as low as 28.18 pg mL^−1^. Moreover, the proposed aptasensor has been successfully applied to determine OTA in actual samples with satisfactory results.

## 1. Introduction

Aptamers (Apts) are oligonucleotide or peptide molecules created by selecting from a large random sequence pool. They are prized for explicitly binding the target and recognized as valuable application tools, including biosensing, bioimaging, and therapeutics [1,2]. In particular, due to the in vitro selection mechanism, aptamers are unprecedented receptors for substances that are difficult to obtain antibodies, such as metal ions and lethal toxins [3]. In addition, aptamers have several advantages over antibodies, such as simple synthesis, uniformity between batches, easy labeling, low cost, and high stability [4,5,6]. A biosensor using an aptamer as a biological identification element was first introduced in 1996 and has been applied to detect various targets, including toxins [7,8,9,10]. Depending on their signal readouts, aptamer-based biosensors (aptasensors) can be categorized as colorimetric, fluorescent, surface-enhanced Raman scattering, or photoelectrochemical sensors. The colorimetric signal is attractive because it makes equipment-free testing possible. Moreover, using a colorimetric aptasensor for on-site detection in solution would eliminate the separation of bonded and unbonded components and significantly simplify the reaction scheme.

Ochratoxin A (OTA), a crucial group of mycotoxins, is mainly produced by two genera: Aspergillus and Penicillium. OTA is the most harmful mycotoxin found extensively in contaminated food among the A, B, and C categories of ochratoxins [11]. It is considered neurotoxic, nephrotoxic, hepatotoxic, carcinogenic, teratogenic, and immunotoxic to humans [11]. In addition, the International Agency for Research on Cancer (IARC) has listed OTA as a possible human carcinogen (2B group) [12,13,14]. Therefore, sensitive detection of OTA has application significance in food surveillance. Although a variety of detection methods have been developed for mycotoxin detection, such as electrochemical detection [14], mass spectrometry [15], chromatography [16,17], enzyme-linked immunosorbent assay [18,19], fluorometry [13,20,21], chemiluminescence [22], and surface-enhanced Raman spectroscopy [23,24], colorimetric OTA aptasensors are attractive because of their low cost and simple and convenient reading without any expensive instruments [25,26].

**Scheme 1:**
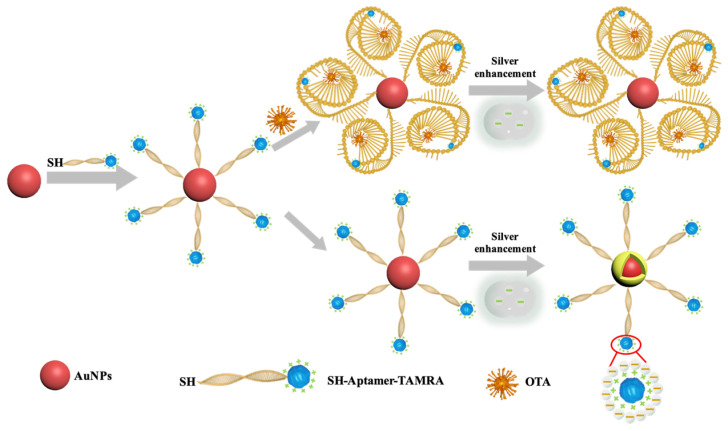
Schematic illustration of the TAMRA-OTA_Apt_@AuNPs–based aptasensor for colorimetric detection of OTA.

Silver enhancement, a procedure for electroless silver deposition, has been used for immunoassays to increase the signal readout. Wang et al. reported an aptamer-based PDMS-gold nanoparticle composite aptasensor and discussed the electrostatic repulsion effects between the negatively charged aptamer and silver lactate on silver enhancements [27]. In previous studies, gold nanoparticles (AuNPs) have been used to catalyze silver reduction on silicon chips [28], glass surfaces [29,30], gold electrodes [31], paper [32], and poly(dimethylsiloxane) (PDMS) [27,33]. In those detection schemes, the AuNP-labeled probes (antibodies or aptamers) were captured on a solid surface, while the unbonded AuNP-labeled probes were removed by washing steps. Thus, washing steps were critical to separate reacted and free AuNPs-labeled probes. During the silver enhancement process, the effective component of the silver enhancement kit is the negatively charged silver lactate. This electron-rich molecule acts as a metal ligand to bind silver ions through coordination [27]. As illustrated in Scheme 1, the AuNPs catalyze silver reduction and act as the nuclei for silver precipitation. In this study, we developed a silver enhancement-based aptasensor for detecting OTA. The 3′-end and 5′-end of the OTA aptamer were tagged with a thiol group (-SH) and a molecular tag tetramethylrhodamine (TAMRA), respectively. As the TAMRA molecule is positively charged, the silver lactate should be attracted and retained around the AuNPs by the TAMRA [34], thus increasing the efficiency of the AuNP-catalyzed silver reduction. In the presence of OTA, the OTA aptamer bound and enwrapped the OTA. The conformation changes of the aptamer formed a barrier to cover TAMRA, thereby interrupting the electrostatic attraction between TAMRA and silver lactate, eventually inhibiting the AuNP-catalyzed silver reduction. The increased barrier of the OTA aptamer complex covering the AuNPs further hindered the interaction between AuNPs and silver lactate. Both effects ultimately reduced the probability of AuNP-catalyzed silver reduction (Scheme 1). With the optimized aptamer concentration and reaction time, the analytical performance of the TAMRA-assisted OTA aptasensor was studied. The potential application of the proposed aptasensor was validated by testing trace OTA spiked in peanuts, corn, and soybean extracts.

## 2. Materials and Methods

### 2.1. Chemicals and Reagents

Tris (2-chloroethyl) phosphate (TCEP), Silver Enhancer Kit were purchased from Sigma-Aldrich (St. Louis, MO, USA). HAuCl_4_ was purchased from Rhawn Reagent (Shanghai, China), trisodium citrate dihydrate was purchased from Kelong Chemical Reagent Factory (Chengdu, China), phosphate-buffered solution (PBS) was purchased from Dingguo Changsheng Biotechnology Co., Ltd. (Beijing, China). OTA was obtained from Cayman Chemical (Ann Arbor, MI, USA). Aflatoxin (AFB1), Fumonisin B1 (FB1), and deoxynivalenol (DON) from Fermentek Ltd. (Jerusalem, Israel). The OTA aptamer (5′GAT CGG GTG TGG GTGGCG TAA AGG GAG CAT CGG ACA-SH-3′) [35], TAMRA tagged OTA aptamer (TAMRA-OTA_Apt_-SH), FAM tagged OTA aptamer (FAM-OTA_Apt_-SH), and ROX tagged OTA aptamer (ROX-OTA_Apt_-SH) were chemically synthesized and purified by Sangon Biotech Co. Ltd. (Shanghai, China). Deionized (DI) water was produced by a water purification system, PURELAB flex (ELGA, High Wycombe, UK).

### 2.2. Apparatus

The UV-vis spectra were recorded on a UV-2250 spectrophotometer (Shimadzu, Kyoto, Japan). The transmission electron microscope technique (TEM, JEOL JEM-2100, Tokyo, Japan) was used to analyze the morphologies of the samples. X-ray photoelectron spectroscopy (XPS) was performed by an ESCALAB 250Xi multi-technique surface analysis system (Thermo Fisher, Waltham, MA, USA). Hydrodynamic size and zeta potential were measured by a Malvern Nano ZS90 Zetasizer (Malvern, Worcestershire, UK).

### 2.3. Preparation of OTA Aptamer Conjugated AuNPs

AuNPs were synthesized by the citrate reduction method, as described previously [36]. Specifically, 2 mL of 1% (m/V) chloroauric acid (HAuCl_4_) was mixed with 198 mL of distilled water in a 500-mL flask. The thoroughly mixed HAuCl_4_ solution was boiled in an oil bath. Then, 4 mL of 1% (m/V) sodium citrate was added to the boiling solution. When the solution turned red and no further color change occurred, the mixture was gradually cooled to room temperature with continuous stirring. The UV-vis absorption spectra of the AuNPs were characterized each time. The obtained AuNPs were stored at 4 °C for future use.

The vial of TAMRA-OTA_Apt_-SH was dissolved in 0.01 M PBS (pH 7.0) to obtain a 100 μM stock solution. The TAMRA-OTA_Apt_-SH disulfide bond was reduced and activated by adding 40 μM tris(2-carboxyethyl) phosphine (TCEP) and mixing for 1 h. Next, 25 μL of 1 μM TAMRA-OTA_Apt_-SH was mixed with 500 μL of AuNPs and incubated at 37 °C for 40 h. Then the mixture was centrifugation at 12,000 rpm for 15 min to collect TAMRA-OTA_Apt_@AuNPs conjugates. The collected TAMRA-OTA_Apt_@AuNPs conjugates were washed three times with 0.01 M PBS (pH 7.0) by centrifugation at 12,000 rpm. Next, the TAMRA-OTA_Apt_@AuNPs conjugates were incubated with 1 μM 6-mercapto-1-hexanol (MCH) at room temperature for 30 min and washed three times with PBS by centrifugation. Then MCH-blocked TAMRA-OTA_Apt_@AuNPs were collected for future use.

### 2.4. TAMRA-OTA_Apt_@AuNP-Based OTA Aptasensors for Colorimetric Detection of OTA

The MCH-blocked TAMRA-OTA_Apt_@AuNPs were incubated with 25 μL of different concentrations of OTA for 30 min at 37 °C. Without washing steps to remove unbonded OTA, the silver enhancement reagent was added into the TAMRA-OTA_APT_@AuNP and OTA mixture to test the AuNPs-catalyzed silver deposition. In brief, 40 μL of silver enhancement solution A and solution B were combined in a 1:1 volumetric ratio and incubated at room temperature for 30 min. Then, the UV-vis absorbances of the solutions were measured.

To evaluate the specificity of the proposed OTA aptasensor, other myotoxins (aflatoxin B1 (AFB1, 100 ng mL^−1^), deoxynivalenol (DON, 100 ng mL^−1^), and fumonisin B1 (FB1, 100 ng mL^−1^)) were analyzed using the same procedure. All experiments were repeated three times.

In addition, peanuts, corn, and soybean were used to test the feasibility of the TAMRA-OTA_Apt_@AuNP-based aptasensor in food sample detection. In brief, the ground non-contaminated food (peanuts, corn, and soybean) were spiked with OTA at different concentrations and mixed in a vortex mixer, according to literature [35]. Then the food powders were treated with extraction solvent (methanol:water = 6:4. *v*/*v*) and centrifuged to collect the supernatant. The extract was passed through a 0.22 µm syringe filter and followed by dilution with PBS buffer to a finally spiked OTA concentration of 1, 5, 10 ng mL^−1^. The TAMRA-OTA_Apt_@AuNP-based OTA aptasensors measured the concentrations.

### 2.5. Statistics Analysis

All experiments were repeated three times over different days. In each replication experiment, the samples were tested triplicates. Data are expressed as the mean ± standard deviation. The effects of oligonucleotide length, molecular tags (TAMRA, FAM, and ROX) on silver deposition and specificity of TAMRA-OTA_Apt_@AuNP in OTA testing were analyzed using analysis of variance (ANOVA) (SPSS software, IBM SPSS Statistics, Chicago, IL, USA). *p*-values less than 0.05 were considered statistically significant.

## 3. Results

### 3.1. Characterization of the TAMRA-OTA_Apt_@AuNP

**Figure 1:**
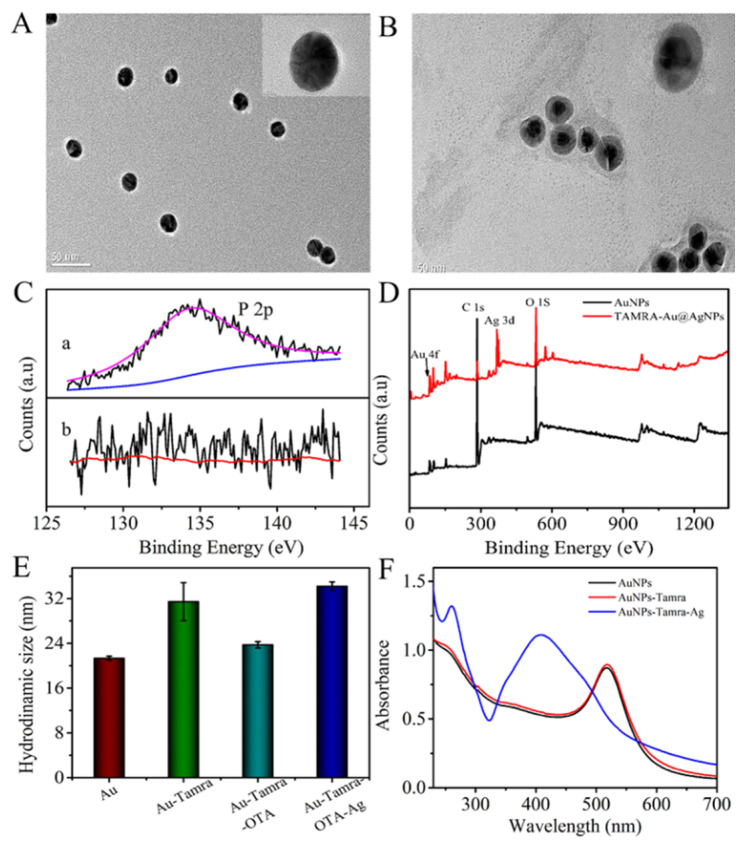
TEM image of (**A**) AuNPs. (**B**) Ag-deposited TAMRA-OTA_Apt_@AuNPs; outer scale bars = 50 nm and inner scale bars = 5 nm. (**C**) The narrow-scan P 2p XPS (a) after and (b) before the aptamer was conjugated on the AuNPs. (**D**) XPS survey of AuNPs, and Ag-deposited TAMRA-OTA_Apt_@AuNPs. (**E**) The hydrodynamic sizes of AuNPs, TAMRA-OTA_Apt_@AuNPs, OTA/TAMRA-OTA_Apt_@AuNPs, Ag-deposited TAMRA-OTA_Apt_@AuNPs. (**F**) UV-vis spectra of AuNPs (black line), TAMRA-OTA_Apt_@AuNPs (red line), and Ag-deposited TAMRA-OTA_Apt_@AuNPs (blue line).

The AuNPs in each reaction were characterized by transmission electron microscopy (TEM). As shown in Figure 1A, the as-prepared TAMRA-OTA_Apt_@AuNPs were uniform spheres with a diameter of ~15 nm. The silver-deposited TAMRA-OTA_Apt_@AuNPs had a dark center (AuNP) and a clear outer part, which indicated the formation of a core-shell Ag@AuNPs structure (Figure 1B).

X-ray photoelectron spectroscopy (XPS) analysis was also conducted to determine the elemental composition of the AuNPs. As shown in Figure 1C, the P 2p peak at 134.5 eV can be observed from the curve of the TAMRA-OTA_Apt_@AuNP-conjugation, indicating a successful attachment of the aptamer on the AuNPs. With the AuNP-catalyzed silver reduction, apart from the C 1s, O 1s, and Au 4f peaks, an Ag 3d characteristic peak can be observed at 368.7 eV (Figure 1D), suggesting the formation of an Ag shell on the surface of the AuNPs.

The hydrodynamic sizes of the AuNPs and the TAMRA-OTA_Apt_@AuNPs were 21.3 ± 0.9 nm and 31.5 ± 3.4 nm, respectively (Figure 1E). The incremental difference in hydrodynamic size is attributed to coated aptamer oligonucleotides extending freely in the solvent. The particle size decreased to 23.7 ± 0.57 nm after incubation with OTA. The size change occurs because the recognition and bonding of aptamer and OTA leads to a conformation change of the oligonucleotides. The addition of the silver enhancement solution caused a significant increase in hydrodynamic size to 34 nm, further suggesting Ag shell formation on the AuNPs (Figure 1E).

The UV-vis absorption spectra of the AuNPs, TAMRA-OTA_Apt_@AuNPs, and silver-deposited TAMRA-OTA_Apt_@AuNPs are shown in Figure 1F. It was found that the adsorption peak of AuNPs is at 520 nm, which is a typic AuNPs’ surface plasmon resonance peak. Conjugating the AuNPs with TAMRA-OTA_Apt_ did not induce a shift in the adsorption peak. However, incubation with a silver enhancement solution led to a new peak at 408 nm, a characteristic Ag peak. The changes in the UV-vis spectra demonstrated the deposition of Ag on the TAMRA-OTA_Apt_@AuNPs. Consequently, the absorbance ratio at 408 nm (Ag) and 520 nm (Au) was used to quantify the AuNP-catalyzed Ag reduction and deposition.

### 3.2. Electrostatic Attraction between TAMRA and Silver Lactate Assists AuNP-Catalyzed Silver Deposition

**Figure 2:**
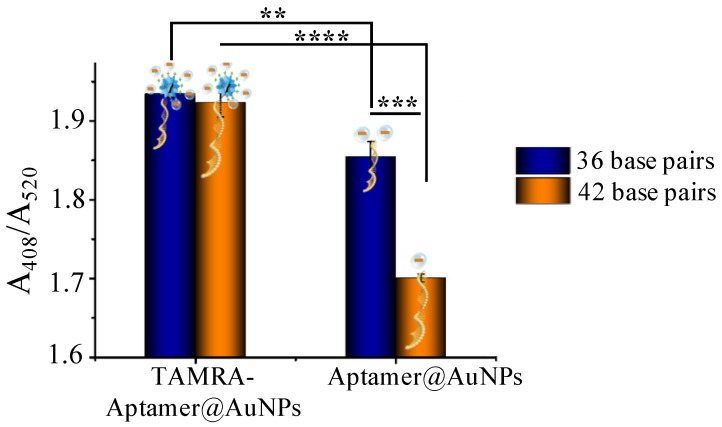
Relative absorbance of silver deposition catalyzed by AuNPs conjugated with aptamers with 36 and 42 base pairs. ** denot *p* < 0.01, *** denot *p* < 0.001, **** denot *p* < 0.0001.

As illustrated in Scheme 1, the reaction principle is that a positively charged tag will enrich the local concentration of silver lactate, thus increasing the probability of AuNP-catalyzed silver reduction and deposition. The silver deposition catalyzed by aptamer@AuNPs and TAMRA-Apt@AuNPs was compared to examine our hypothesis. Oligonucleotides with 36 and 42 base pairs were tested (36 base pair: 5′-TAMRA-GAT CGG GTG TGG GTGGCG TAA AGG GAG CAT CGG ACA-SH-3′; 42 base pairs: 5′-TAMRA-ATC TAC GAA TTC ATC AGG GCT AAA GAG TGC AGA GTT ACT TAG-SH-3′). As shown in Figure 2, the TAMRA-Apt@AuNPs, with 36 base pairs and 42 base pairs, have significantly higher efficiency in catalyzing Ag deposition than the Apt@AuNPs. In addition, when the aptamer length increased from 36 base pairs to 42 base pairs, the capability of the aptamer@AuNPs catalysis Ag deposition decreased dramatically (Apt_36_@AuNPs vs. Apt_42_@AuNPs: 1.86 ± 0.02 vs. 1.69 ± 0.01, *p* = 0.0002); meanwhile, there was no significant difference between the efficiency of the TAMRA-Apt_36_@AuNPs and TAMRA-Apt_42_@AuNPs (TAMR-Apt_36_@AuNPs vs. TAMRA-Apt_42_@AuNPs: 1.93 ± 0.01 vs. 1.92 ± 0.02, *p* = 0.51), suggesting that TAMRA can mitigate the impact of oligonucleotide length on Ag deposition. The long aptamers may hinder the access of the AuNPs to the silver deposition elements in the aptamer-AuNPs. However, the silver lactate trapping capabilities of the TAMRA-Apt@AuNPs may alleviate the aptamer length-caused signal decrease.

**Figure 3:**
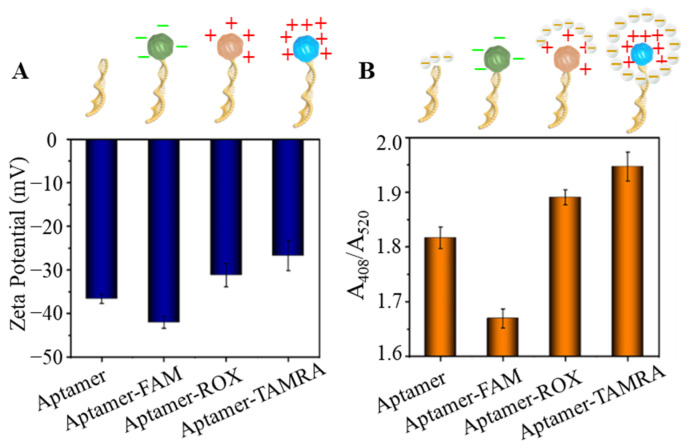
(**A**) Zeta potential of aptamer@AuNPs tagged with different molecular tags. (**B**) relative absorbance (A_408_/A_520_) changes induced by aptamer@AuNPs tagged with different molecular tags. FAM: fluorescein amidites, ROX: 6-carboxy-x-rhodamine, TAMRA: tetramethylrhodamine.

To further examine our hypothesis, molecular tags, fluorescein amidites (FAM) and 6-carboxy-x-rhodamine (ROX), were used to label OTA aptamer (5′-FAM-GAT CGG GTG TGG GTG GCG TAA AGG GAG CAT CGG ACA-SH-3′, 5′-ROX GAT CGG GTG TGG GTG GCG TAA AGG GAG CAT CGG ACA-SH-3′). The molecular tag labeled OTA aptamers were conjugated on AuNPs (FAM-OTA_Apt_@AuNPs, and ROX-OTA_Apt_@AuNPs) to test the capability of the AuNPs on Ag reduction. The labeling of the molecular tag (TAMRA, FAM, and ROX) did not induce the aggregation of AuNPs in PBS (pH 7.0), as no obvious UV-vis AuNPs’ surface plasmon resonance peak (520 nm) shift was observed. First, the overall charge of the molecular tag OTA_Apt_@AuNPs was characterized by a Malvern Nano ZS90 Zetasizer. As shown in Figure 3A, the Zeta potential of Apt@AuNPs is −36.6 ± 1.1 mV. While the molecular tag of FAM on the OTA aptamer changes the overall Zeta potential of FAM-OTA_Apt_@AuNPs to −42.1 ± 1.4 mV, suggesting an increase of negative charging. However, the overall Zeta potential of ROX-OTA_Apt_@AuNPs, and TAMRA-OTA_Apt_@AuNPs were −31.2 ± 1.6 mV, and −26.7 ± 3.5 mV, respectively. The reduced absolute negative charge revealed by the Zeta potential characterization results suggest that molecular tags can change the overall charge of the Apt@AuNPs, potentially impacting the catalyzation of the AuNPs. Next, the effect of the charges of the molecular tag on AuNP-catalyzed silver reduction and deposition was quantified. As shown in Figure 3B, the highest silver disposition resulted from the least negative conjugate (TAMRA-OTA_Apt_@AuNPs), and the weakest silver deposition was from the conjugate with the most significant negative value (FAM-OTA_Apt_@AuNPs). The relationship between the surface charge of the aptamer@AuNP conjugates and the level of AuNP-catalyzed silver deposition, as demonstrated in Figure 2 and Figure 3A,B, suggests that a charged molecular tag can enhance silver deposition by attracting and retaining the silver deposition element (silver lactate) around the AuNPs to facilitate the catalysis. The charged tag-enhanced Ag deposition is essential to construct sensitive aptasensors with long oligomer aptamers. Therefore, TAMRA was selected to construct an OTA aptasensor.

### 3.3. Optimization of Analytical Parameters

**Figure 4:**
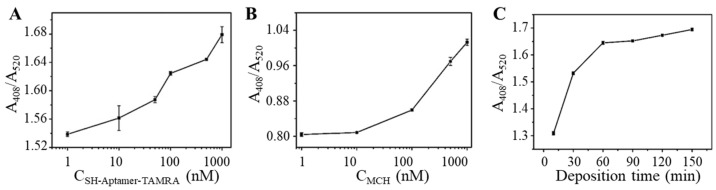
The optimization of (**A**) Concentration of TAMRA-OTA_Apt_-SH; (**B**) Concentration of 6-mercapto-1-hexanol (MCH); (**C**) Silver deposition time. (*n* = 9).

To achieve sensitive and fast OTA detection, parameters involved in the detection process were optimized to increase sensitivity and maximize the efficiency of the aptasensor. First, the concentration of TAMRA-OTA_Apt_-SH in preparing TAMRA-OTA_Apt_@AuNPs conjugates was optimized. It can be seen from Figure 4A that as the concentration of TAMRA-OTA_Apt_-SH increased from 1 to 1000 nM, the absorbance ratio of A_408_/A_520_ response gradually increased. Therefore, 1000 nM TAMRA-OTA_Apt_-SH was used to construct the OTA_Apt_@AuNP-based aptasensor. Next, MCH was used to block the unoccupied sites on the TAMRA-Aptamer@AuNP surface. It was reported that MCH could prevent AuNP aggregation [37], thus ensuring the stability of AuNP solutions. As shown in Figure 4B, TAMRA-Apt@AuNP passivated by 1 μM MCH delivered the highest absorbance ratio of A_408_/A_520_, suggesting an improved Ag deposition. Finally, the Ag deposition time was optimized. As shown in Figure 4C, the absorbance ratio of A_408_/A_520_ gradually increased as the deposition time increased from 15 min to 60 min. Further extension of the deposition time did not result in signal enhancement. Thus, the Ag deposition time was set as 60 min.

### 3.4. Analytical Performance of TAMRA-OTA_Apt_@AuNP-Based OTA Aptasensor

**Figure 5:**
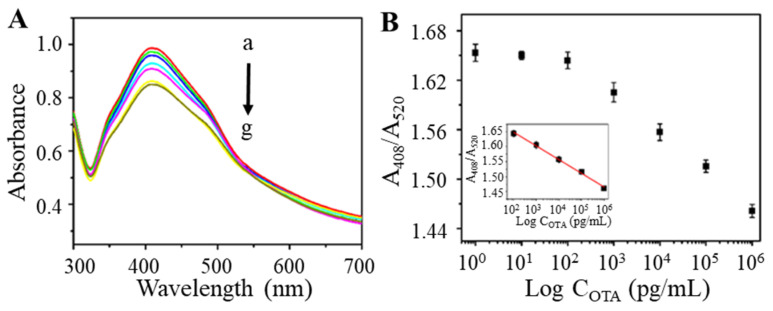
(**A**) The UV-vis absorption spectra corresponding to various OTA concentrations (from a to g: 1, 10, 10^2^, 10^3^, 10^4^, 10^5^, and 10^6^ pg mL^−1^) based on TAMRA-OTA_Apt_@AuNPs. (**B**) The relationship between the relative absorption and the concentrations of OTA. Inset: the plot of relative absorption versus OTA concentration ranging from 10^2^ to 10^6^ pg mL^−1^.

With the optimized parameters, the TAMRA-OTA_APT_@AuNP-conjugates were prepared for OTA testing. The TAMRA-OTA_APT_@AuNP was mixed with different concentrations of OTA and incubated for 30 min at 37 °C. As shown in Figure 5A, the UV-vis absorption gradually decreased as the concentration of OTA increased. The calibration curve of absorbance intensity (A_408_/A_520_) vs. OTA concentrations is shown in Figure 5B. The absorbance ratio of A_408_/A_520_ was proportional to the logarithm of OTA concentration and within a concentration range of 1 × 10^2^ to 1 × 10^6^ pg mL^−1^. Therefore, we obtained a linear equation expressed as A_408_/A_520_ = 1.739 − 0.04456 log C_OTA_ with the correlation coefficient R^2^ = 0.995. According to reported colorimetric aptasensors [38,39], the limit of detection (LOD) of 28.18 pg mL^−1^ was calculated as 3 times the standard deviation (s.d.) of the blank sample signal (s.d. = 2.48 × 10^−2^) divided by the slope of the regression equation (slope = −0.04456). On this basis, a colorimetric detection method for OTA has been successfully constructed. The merits and novelty of this work lie in the charged molecular tag, TAMRA, that enriches the local silver lactate concentration to facilitate AuNP-catalyzed silver reduction to produce visual colorimetric changes. The analytical performance of the TAMRA-OTA_APT_@AuNP-based aptasensor was compared with previously reported colorimetric OTA biosensing (Table 1). The proposed TAMRA-OTAAPT@AuNPs-based aptasensor achieved a broader working range and comparable detection limit. In addition, the solution reaction system combined with the AuNP-catalyzed silver enhancement eliminates the need to separate bound and unbound molecules in the reaction system, thus significantly simplifying the overall reaction procedure.

**Figure 6:**
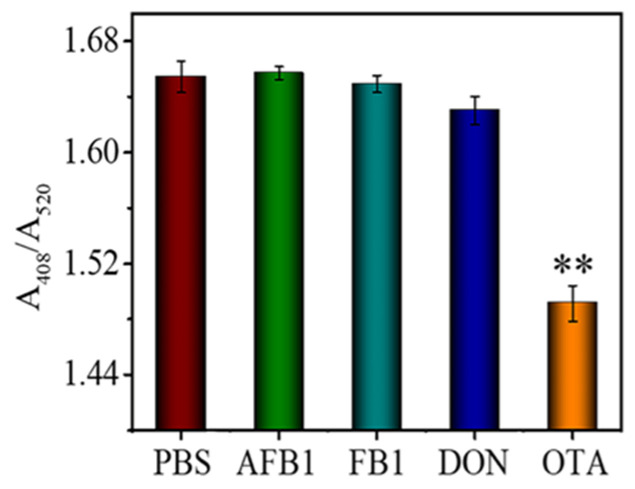
The response of the aptasensor to 100 ng mL^−1^ of AFB1, FB1, DON, and OTA. ** denotes the *p* < 0.01.

Specificity is of great importance for sensor applications. To investigate the specificity of the developed aptasensor, AFB1, DON, and FB1 were analyzed as interfering substances (Figure 6). The more robust silver enhancement-induced colorimetric change was observed from FB1, AFB1, and DON samples. This is because the TAMRA-OTA_Apt_ immobilized on the AuNPs stretched in the solution, while the TAMRA molecules attracted silver lactate to increase the probability of AuNP-catalyzed Ag deposition. The OTA sample gave the lowest absorbance (A_408_/A_520_) change (Figure 6; *p* < 0.01). The results demonstrate that the colorimetric aptasensor has good specificity and selectivity for OTA detection.

To investigate the applicability of the proposed method, trace amounts of OTA were spiked into ground peanuts, corn, and soybean samples; the food extracts were prepared and measured via the proposed colorimetric aptasensor. Table 2 shows that the detection of spiked OTA (1, 5, and 10 ng mL^−1^) in peanuts, corn, and soybean had recovery rates ranging from 99.53–104.67% with a coefficient of variation (CV) of 9.97–11.04%, 99.47–106.43% with the CV of 6.34–14.45%, and 97.03–99.95% with the CV of 5.87–11.27%, respectively. The successful detection trace of OTA in peanuts, corn, and soybean samples further demonstrated the potential of the proposed aptasensor in food sample analysis. However, the impact of reaction pH on silver deposition induced by different molecule tags was not systematically investigated. In addition, the limitation of the AuNP-based aptasensor is the narrow working pH range, which should be considered in real sample testing.

## 4. Conclusions

In summary, we report a sensitive OTA aptasensor constructed from TAMRA-OTA_Apt_@AuNPs. The positive TAMRA tag improved the signal readout by attracting and enriching the silver lactate around the AuNPs to increase the probability of AuNP-catalyzed silver reduction and deposition. This strategy is essential for long aptamers with large base pair numbers. The TAMRA-OTA_Apt_@AuNP-based OTA aptasensor exhibited excellent sensing performance towards OTA with a linear range of 1 × 10^2^ to 1 × 10^6^ pg mL^−1^ and a low detection limit 28.18 pg mL^−1^. In addition, spiked trace amounts of OTA were successfully detected in peanuts, soybean, and corn, demonstrating the potential of this technique for food sample analysis.

## Figures and Tables

**Table 1:** Comparison of analytical performance of colorimetric OTA aptasensors.

Sensing Materials	Working Range	Limit of Detection (ng/mL)	Reference
Au/Fe_3_O_4_	10–10^5^	1.15	[40]
AuNPs	8.06–251.88	8.06	[41]
AuNPs	32–1024	20	[42]
MnO_2_-based nanozyme	1.25–250 nM	0.069 nM	[43]
Au/Fe_3_O_4_	0.5–100	30 pg/mL	[35]
AuNPs	0.05–50 ng/mL	0.009 ng/L	[44]
Au@Fe_3_O_4_	0.5–80 ng/mL	0.15 ng/mL	[26]
AuNPs	1 × 10^2^ to 1 × 10^6^ pg mL^−1^	28.18 pg/mL	This work

**Table 2:** Detection of trace amount of OTA in food extractions.

Food Sample	Spiked OTA (ng mL^−1^)	Detected OTA (ng mL^−1^)	Recovery (%)	CV (%)
Peanuts	1	1.05 ± 0.11	104.67	10.50
	5	4.98 ± 0.50	99.53	9.97
	10	10.44 ± 1.15	104.42	11.04
Corn	1	1.06 ± 0.11	106.33	10.68
	5	5.32 ± 0.34	106.43	6.34
	10	9.95 ± 1.34	99.47	13.45
Soybean	1	0.99 ± 0.11	99.83	11.27
	5	4.85 ± 0.28	97.03	5.87
	10	10.00 ± 0.95	99.95	9.54

## Data Availability

The data in the current study are available from the corresponding authors upon reasonable request.
